# Synthesis, phloem mobility and induced plant resistance of synthetic salicylic acid amino acid or glucose conjugates

**DOI:** 10.1002/ps.7112

**Published:** 2022-08-26

**Authors:** Benoit Guichard, Hanxiang Wu, Sylvain La Camera, Richa Hu, Cécile Marivingt‐Mounir, Jean‐François Chollet

**Affiliations:** ^1^ Institut de Chimie des Milieux et des Matériaux de Poitiers (IC2MP), Unité Mixte de Recherche CNRS 7285 Université de Poitiers Poitiers France; ^2^ State Key Laboratory for Biology of Plant Diseases and Insect Pests, Institute of Plant Protection Chinese Academy of Agricultural Sciences Beijing China; ^3^ Laboratoire Écologie & Biologie des Interactions, Unité Mixte de Recherche CNRS 7267 Université de Poitiers Poitiers France; ^4^ School of Nuclear Technology and Chemistry & Biology Hubei University of Science and Technology Xianning China

**Keywords:** salicylic acid, integrated pest management, defense elicitor, vectorization strategy, maize stalk rot, southern corn leaf blight

## Abstract

**BACKGROUND:**

The growing demand for food, combined with a strong social expectation for a diet produced with fewer conventional agrochemical inputs, has led to the development of new alternatives in plant protection worldwide. Among different possibilities, the stimulation of the plant innate immune system by chemicals represents a novel and promising way. The vectorization strategy of an active ingredient that we previously developed with fungicides can potentially extend to salicylic acid (SA) or its halogenated analogues.

**RESULTS:**

Using the click chemistry method, six new conjugates combining SA or two mono‐ or di‐halogenated analogues with L‐glutamic acid or β‐D‐glucose *via* a 1,2,3‐triazole nucleus have been synthesized. Conjugate **8a**, which is derived from SA and glutamic acid, showed high phloem mobility in the *Ricinus* model, similar to that of SA alone despite a much higher steric hindrance. *In vivo* bioassays of the six conjugates against two maize pathogenic fungi *Bipolaris maydis* and *Fusarium graminearum* revealed that, unlike SA, the amino acid conjugate **8a** with good phloem mobility exerted a protective effect not only locally at the application site, but also in distant stem tissues after foliar application. Moreover, compounds **8a** and **8b** induced up‐regulation of both defense‐related genes *ZmNPR1* and *ZmPR1* similar to their parent compounds upon challenge inoculation with *B. maydis*.

**CONCLUSION:**

The vectorization of salicylic acid or its halogenated derivatives by coupling them with an α‐amino acid can be a promising strategy to stimulate SA‐mediated plant defenses responses against pathogens outside the application site. © 2022 The Authors. *Pest Management Science* published by John Wiley & Sons Ltd on behalf of Society of Chemical Industry.

## Graphical abstract

Vectorization of salicylic acid by coupling it with an α‐amino acid may be a promising strategy to stimulate SA‐mediated plant defense responses against pathogens.

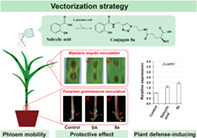

## 1 INTRODUCTION

Over the last seven decades, the advent of organic chemistry and the emergence of plant protection products contributed to an increase in crop yields that was previously technically impossible. However, undesirable effects of agrochemicals on human health and the environment have soon become apparent.^1^, ^2^ In addition, repeated use of single‐site acting compounds caused pesticide resistance in pests, which in turn resulted in raising the economic costs of agrochemical use.^3^ Fungicide resistance in some pathogens was detected after only 2 years of product introduction.^4^ Hence, there is an urgent necessity for the innovation of green pest management technologies,^5^, ^6^ including new and safer agrochemicals.^7^

In the long‐term competitive host‐pathogen coevolution, plants have formed multilevel defense mechanisms to combat pathogen infection, including increasing cell wall strength, synthesizing antibacterial secondary metabolites, expressing pathogenesis‐related proteins and hypersensitive response.^8^, ^9^ One particular inducible systemic immune response, known as systemic acquired resistance (SAR), mediates long‐lasting broad‐spectrum resistance to a wide range of pathogens in uninfected tissue to prevent second infection.^10^, ^11^ At present, the development of certain natural and synthetic chemical inducers that can trigger similar plant defense systems has become an important way to promote green plant protection.^12^, ^13^, ^14^

Exogenous application of defense‐related hormone salicylic acid (SA) and SA analogs has been reported to induce SAR‐like responses.^12^ SA showed fungistatic effect for limiting the growth of parasitic fungi *in vitro* ^15^ or *in vivo* when it is brought exogenously to the plants.^16^ However, it is rapidly metabolized,^17^, ^18^ which can limit its practical use. To overcome this problem, synthetic analogues have been proposed such as acibenzolar‐S‐methyl^19^ and 2,6‐dichloroisonicotinic acid (INA).^20^, ^21^, ^22^ The strategy proposed here is different and involves the prodrug concept. In previous works, it was shown that by coupling an active ingredient to a nutrient, mainly an α‐amino acid, the resulting conjugates could be recognized and manipulated by the plasma membrane transporters of the sieve element‐companion cell complex and thus be translocated to new growth *via* the phloem sap.^23^, ^24^, ^25^, ^26^ The nutrient promoiety, amino acid or sugar,^27^, ^28^ as well as the structure of the spacer arm that connects the active substance to the nutrient,^29^ play key roles in the rate of conjugate translocation and bioactivation. In some cases, it has been shown that the conjugates released the active substance progressively and thus exhibited prodrug behavior.^24^, ^25^, ^30^

The objective of this work was to synthesize new conjugates associating salicylic acid or two mono‐ or dichlorinated analogues with a nutrient promoiety. Regarding the latter, L‐glutamic acid was first chosen because its additional carboxylic acid function was necessary to ensure the binding with the active ingredient while keeping free α‐amino acid function,^31^ thus allowing the recognition by the amino acid transporters of the plasma membrane. In order to study the influence of the nutrient on the biological properties, a similar series with β‐D‐glucose was also prepared. The binding between salicylic acid or its halogenated analogues and the nutrient was achieved by a click chemistry procedure. The phloem systemicity of the six resulting conjugates was then estimated using the *Ricinus* model. Finally, the defense‐inducing activities of the resulting conjugates were evaluated on maize seedlings inoculated with two pathogens that are responsible for southern corn leaf blight and maize stalk rot, respectively. The expression profiles of two defense‐related genes *ZmNPR1* and *ZmPR1* were also studied after treatment with the conjugates.

## 2 MATERIALS AND METHODS

### 2.1 Chemicals

The different solvents used for the organic syntheses were purchased from Acros Organics (Fisher Scientific SAS, Illkirch, France). The reagents used were purchased from Acros Organics (2,3,4,6‐tetra‐O‐acetyl‐alpha‐D‐glucopyranosyl bromide, 5‐chlorosalicylic acid, copper sulfate pentahydrate, sodium ascorbate, sodium azide), TCI Europe N.V. (Paris, France; 2‐bromoethylamine hydrobromide, 3,5‐dichlorosalicylic acid, (1‐(3‐dimethylaminopropyl)‐3‐ethylcarbodiimide hydrochloride (EDCl), N‐Boc‐L‐glutamic acid 1‐tert‐butyl ester, propargylamine), Alfa Aesar (Thermo Fisher GmbH, Kandel, Germany; 4‐dimethylaminopyridine (DMAP), acetic anhydride, Amberlite IRN 77, sodium acetate), Sigma Aldrich (Merck KGaA, Darmstadt, Germany; salicylic acid, aspirin, 2‐(N‐morpholino)ethanesulfonic acid monohydrate [MES buffer]), Solarbio (Solarbio Science & Technology Co., Ltd., Beijing, China; surfactant Silwet‐L77, Tween‐20).

### 2.2 Synthesis

Some reactions were carried out under nitrogen. All reactions were monitored by TLC analysis using Merck silica gel 60F‐254 thin‐layer plates. Column chromatography was carried out on silica gel Merck 60 (0.015–0.04 mm). Melting points were determined on a Büchi B‐540 melting point apparatus and are uncorrected. ^1^H and ^13^C NMR spectra were performed in CDCl_3_ or DMSO‐d_6_ using a Bruker AVANCE 400 MHz or a Bruker Ultrashield™ 500 Avance NEO spectrometer, respectively at 400 or 500 MHz frequencies for ^1^H experiments and 101 or 126 MHz for ^13^C experiments. DEPT (90 and 135) and 2D experiments (^1^H—^13^C and ^1^H—^1^H) were used to confirm the NMR peak assignments. Chemical shifts are reported as ∂ values in parts per million (ppm) relative to tetramethylsilane (TMS) as internal standard and coupling constants (*J*) are given in hertz (Hz). The following abbreviations are used to describe peak patterns when appropriate: s (singlet), d (doublet), t (triplet), q (quartet), m (multiplet). High‐resolution mass spectra were obtained on a Bruker Q‐TOF Impact HD spectrometer using an electrospray ionization source (ESI).

### 2.3 Plant material and fungal strain

#### 2.3.1 Systemicity test

Castor bean seeds (*Ricinus communis* L. cv Sanguineus), obtained from Zibo Academy of Agricultural Sciences (Shandong, China) were placed in wet cotton wool for 24 h at 27 °C ± 1 °C prior to sowing in vermiculite watered with tap water. Seedlings were grown in a humid atmosphere (80% ± 5%) at 28 °C ± 1 °C.

#### 2.3.2 Activity on plant defense response

Two pathogens were used in this study. *Bipolaris maydis*, the causal agent of southern‐corn leaf blight disease, was isolated in Gongzhulin, Jilin, China. *Fusarium graminearum* is a soil‐born pathogen that causes stalk rot of maize, which was also isolated in Gongzhulin, Jilin, China. *B. maydis* and *F. graminearum* were grown on oat‐meal agar (OA) and potato dextrose agar (PDA) medium plates at 28 °C before collecting conidia for inoculation, respectively. Maize B73 inbred line was grown in Conviron growth chamber at 28 °C with a 16 h photoperiod for 2–3 weeks.

### 2.4 Phloem sap collection and analysis

The sap collection method was similar to that previously described.^32^ The phloem sap was analyzed using an Agilent Technologies 1260 high‐performance liquid chromatography after dilution with UHQ grade water (1:9; v/v). An Agilent SB‐C18 reversed‐phase column (length 250 mm, internal diameter 4.6 mm, 5 μm) was used at a flow rate of 0.8 mL min^−1^ in accordance with the procedure set out in Table 1. The injection volume was 10 μL.

**Table 1:** Chromatographic data for tested products

Product	Mobile phase (gradient)				
	Time (min)	Methanol (%)	Water +0.1% TFA (%)	UV detection (nm)	Retention time (min)
	*t* = 0	30	70	234	
**SA**	*t* = 16	85	15		15.7
**8a**	*t* = 18	85	15		8.4
**9a**	*t* = 20	30	70		9.5
	*t* = 23	30	70		
	*t* = 0	45	55	230	
**5‐ClSA**	*t* = 16	90	10		16.3
**8b**	*t* = 18	90	10		8.2
**9b**	*t* = 20	45	55		9.5
	*t* = 23	45	55		
	*t* = 0	55	45	240	
**3,5‐diClSA**	*t* = 16	90	10		17.1
**8c**	*t* = 18	90	10		9.0
**9c**	*t* = 20	55	45		10.4
	*t* = 23	55	45		

### 2.5 Evaluation of activity on plant defense response

The *in vitro* fungicidal activities of SA conjugates against *B. maydis* were evaluated on OA plates. The compound was dissolved in 1 mL of absolute ethanol and diluted with 5 mL of sterile water containing 0.1% Tween 80 at the concentration of 10 mm. Then, 1 mL of mixture solution was added to 9 mL OA medium. The final concentration of each compound was 1 mm in Petri dishes. The same ethanol solution without tested compound was served as control. The plates were inoculated with agar plugs (5 mm diameter) from a 6‐day‐old growing colony of *B. maydis* strain and incubated in the dark at 28 °C. The colony diameter was measured 6 days after inoculation. There were three replications for each treatment.

Maize infection assays were performed using inbred line B73. Tested conjugate was sprayed (2 mL per plant) on the leaves of maize seedling at a concentration of 1 mm. The conjugate was solubilized in ethanol (5% of final volume of application solution) and diluted with 10 mm MES buffer solution (pH 5.0) containing 0.15% Silwet‐L77. The same application solution without tested compound served as a control. After 2 days, the two pathogens were individually inoculated to pretreated maize seedlings. Detached‐leaf‐spotting assays were used for the inoculation of *B. maydis* according to a previous described method.^33^ The conidial suspension of *B. maydis* was prepared at a final concentration of 1 × 10^5^ conidia/mL by flooding 12‐day‐old *B. maydis* culture plates with sterile distilled water containing 0.1% (v/v) Tween‐20. Two droplets (10 μL each) of conidial suspension were spotted on to a detached maize leaf (10 cm in length) from conjugate pretreated plants. The inoculated leaf was kept in a Petri dish that contained 0.1% 6‐benzylaminopurine sterile water and then transferred to a chamber at 28 °C and 90% relative humidity for 4 days. The lesion size was determined using ImageJ software after photographing. Artificial inoculation of *F. graminearum* on maize seedling stem was performed as described previously by Sun *et al*.^34^ 10 μL of conidial suspensions of *F. graminearum* (1.0 × 10^6^ conidia/mL in 0.1% Tween‐20) was dropped to the wounded point of seedling stem using a pipette. The seedlings were maintained under a condition at 28 °C and 90% relative humidity for 3 days without moving. Disease severity assessments were performed using a scale ranging from 1 to 5 described by Sun *et al*.^34^ The disease severity (%) = ∑ (Number of plants in that rating × rating)/(total number of plants assessed × maximum rating) × 100. There were three replications for each treatment.

For the analysis of defense‐related gene expression, maize seedlings were also pretreated with 1 mm SA conjugates following foliar application. Plants treated with blank application solution were used as control. The expression of the non‐expressor of pathogenesis‐related genes 1 (*NPR1)* and the defense marker gene pathogenesis‐related‐1 (*PR‐1*) was measured using quantitative real‐time PCR (qRT‐PCR) with or without pathogen challenge. In the absence of challenge, the leaves at the same positions of seedlings were harvested for RNA extraction 24 h after chemical treatment. In *B. maydis* challenged group, the abovementioned conidial suspension of *B. maydis* (1 × 10^5^ conidia/mL) was sprayed on the maize plantlets (0.5 mL per plant) 48 h after chemical treatment, then the inoculated leaves at the same positions were collected 24 h post‐inoculation. Total RNA was extracted using Plant Total RNA Isolation Kit (FOREGENE, Chengdu, China). First‐strand cDNA was subsequently synthesized using a One‐Step gDNA Removal and cDNA Synthesis SuperMix (TransGen Biotech). Primers for *ZmNPR1* were 5′‐ACAAACTGCTGATGGTGATACG‐3′ (forward primer) and 5′‐GCAACTTCACAAGCTCAACATC‐3′ (reverse primer). Primers for *ZmPR1* were 5′‐CCACTACGGGGAGAACATCT‐3′ (forward primer) and 5′‐AGTTGCAGGTGATGAAGACG‐3′ (reverse primer). qRT‐PCR was performed by a 7500 Real‐Time PCR System (Applied Biosystems, CA, USA) using the kit of RealStar Green Fast Mixture (Genestar). The Expression values were normalized to the expression of *ACTIN1* using the primers *Actin1*‐F (5′‐GATTCCTGGGATTGCCGAT‐3′) and *Actin1*‐R (5′‐TCTGCTGCTGAAAAGTGCTGAG‐3′). The transcription level was calculated using the 2^‐ΔΔCT^ method.^35^ The experiments were repeated three times.

### 2.6 Physicochemical properties

Physicochemical properties and descriptors were predicted using ACD/Labs Percepta 2015 release (Build 2726) software from Advanced Chemistry Development, Inc. (Toronto, Canada). The calculated properties (Table 2) were chosen according to their influence on passive membrane transport in plants.^36^

**Table 2:** Structures, chemical descriptors and physicochemical properties of the studied compounds computed with ACD/Labs Percepta 2015 release (Build 2726) software

Product name/number	Structure	MW	HBD	HBA	Log D (pH 5.0)	Log D (pH 8.0)	FRB	PSA (Å^2^)	Lipinski's rule of five violation	Veber rules violation
Salicylic acid (SA)	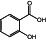	138.12	2	3	0.37	−1.68	1	57.53	0/4	0/2
5‐chlorosalicylic acid (5‐ClSA)	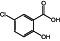	172.57	2	3	0.82	−0.87	1	57.53	0/4	0/2
3,5‐dichlorosalicylic acid (3,5‐diClSA)	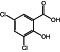	207.01	2	3	1.02	−0.22	1	57.53	0/4	0/2
**8a**	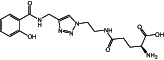	390.39	6	11	−0.66	−0.85	10	172.46	2/4	1/2
**8b**	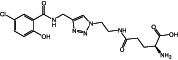	424.84	6	11	0.26	−0.37	10	172.46	2/4	1/2
**8c**	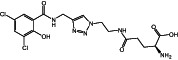	459.28	6	11	0.97	0.05	10	172.46	2/4	1/2
**9a**	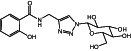	380.35	6	11	−0.62	−0.80	5	170.19	2/4	1/2
**9b**	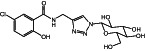	414.80	6	11	0.34	−0.44	5	170.19	2/4	1/2
**9c**	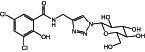	449.24	6	11	1.09	−0.42	5	170.19	2/4	1/2

The interpretation of the computed properties to predict crossing a biological membrane is given according to Lipinski's rule of five (*MW* ≤ 500 Da; *HBD* ≤ 5; *HBA* ≤ 10; *Log P* ≤ 5.0) and to Veber rule (*FRB* ≤ 10; *PSA* ≤ 140 Å^2^). At biological pHs (from 5.0 to 8.0), the carboxylic acid functions of salicylic acid and its chlorinated analogues are predicted to be totally in their dissociated form (pKa = 3.0 ± 0.1 for SA, 2.6 ± 0.1 for 5‐ClSA and 2.0 ± 0.1 for 3,5‐diClSA) and amino acid conjugates **8a‐8c** are predicted to be under their zwiterrionic form.

FRB, free rotatable bonds; HBA, number of hydrogen bond acceptors; HBD, number of hydrogen bond donors; MW, molecular weight; PSA, polar surface area.

### 2.7 Statistical analyses

To examine the *in vitro* activity of the different products on the radial growth of *B. maydis*, three assays were performed for each compound. The Kruskal‐Wallis nonparametric test coupled with Dunn's multiple comparison test was used to assess statistically significant differences between the control and the nine treated sets, assuming significance at *P* ≤ 0.05.

To evaluate the protective effect of parent compounds and the conjugates against *B. maydis* and *F. graminearum* on maize, the assays using 12 detached leaves or three pots with five plants were performed for every product, respectively. A one‐way ANOVA followed by Tukey's HSD test was performed to assess statistically significant differences between the control and the treated sets or among the different compounds tested for *B. maydis*, assuming significance at *P* ≤ 0.05.

To investigate the expression of the defense‐related genes *ZmNPR1* and *ZmPR1* in maize leaves, a representative experiment with three samples was selected from three replicates showing the same trend. A one‐way ANOVA followed by a Dunnett's multiple comparison test was performed to assess statistically significant differences between the control and the treated sets.

## 3 RESULTS AND DISCUSSION

### 3.1 Synthesis of L‐glutamic acid and β‐D‐glucose conjugates

In previous work, we described the three‐step synthesis of conjugates that associated fenpiclonil, a fungicide from the phenylpyrrole family, with a nutrient that could be an amino acid or a sugar. While an active transporter recognizes these compounds, they are manipulated in different ways. The fenpiclonil‐glutamic acid conjugate showed a much more favorable phloem mobility than fenpiclonil‐glucose conjugate.^27^ Similarly, the structure of the spacer arm that connects fenpiclonil to the nutrient greatly influences the phloem mobility of the conjugate.^29^ Surprisingly, the latter has proven to be an excellent tool to study the properties of sucrose carriers in plants.^28^

In this work, we have developed a four‐step method for the synthesis of new conjugates associating salicylic acid or two chlorinated analogues with L‐glutamic acid or β‐D‐glucose: (i) the first step consisted of obtaining azides from glutamic acid or glucose whose reactive functions were protected to avoid unwanted side reactions in the following stages; (ii) the second step allowed to obtain propargyl derivatives from salicylic acid and its halogenated analogues that will be coupling partners for subsequent click chemistry reactions; (iii) in the third step, the two derivatives obtained during the previous stages were condensed by a click chemistry process to give the protected conjugates with a spacer arm containing a 1,2,3‐triazole ring and finally, (iv) the α‐amino acid function of glutamic acid or hydroxyl groups of glucose were deprotected to give the desired conjugates. The series of six compounds thus obtained is coherent for further structure–activity relationship studies: the active part of the conjugate is represented by salicylic acid or by two mono‐ or dichlorinated analogues, the part related to the vectorization of the conjugate is a nutrient which can be an α‐amino acid or a sugar, while in all cases the spacer arm is linked to the active part of the molecule by an amide bond and includes a 1,2,3‐triazole ring in its structure.

#### 3.1.1 *Synthesis of azido derivatives from protected amino acid or sugar (Figs* 1 *and* 2 *; compounds* ***2*** *and* ***3*** *)*

**Figure 1:**
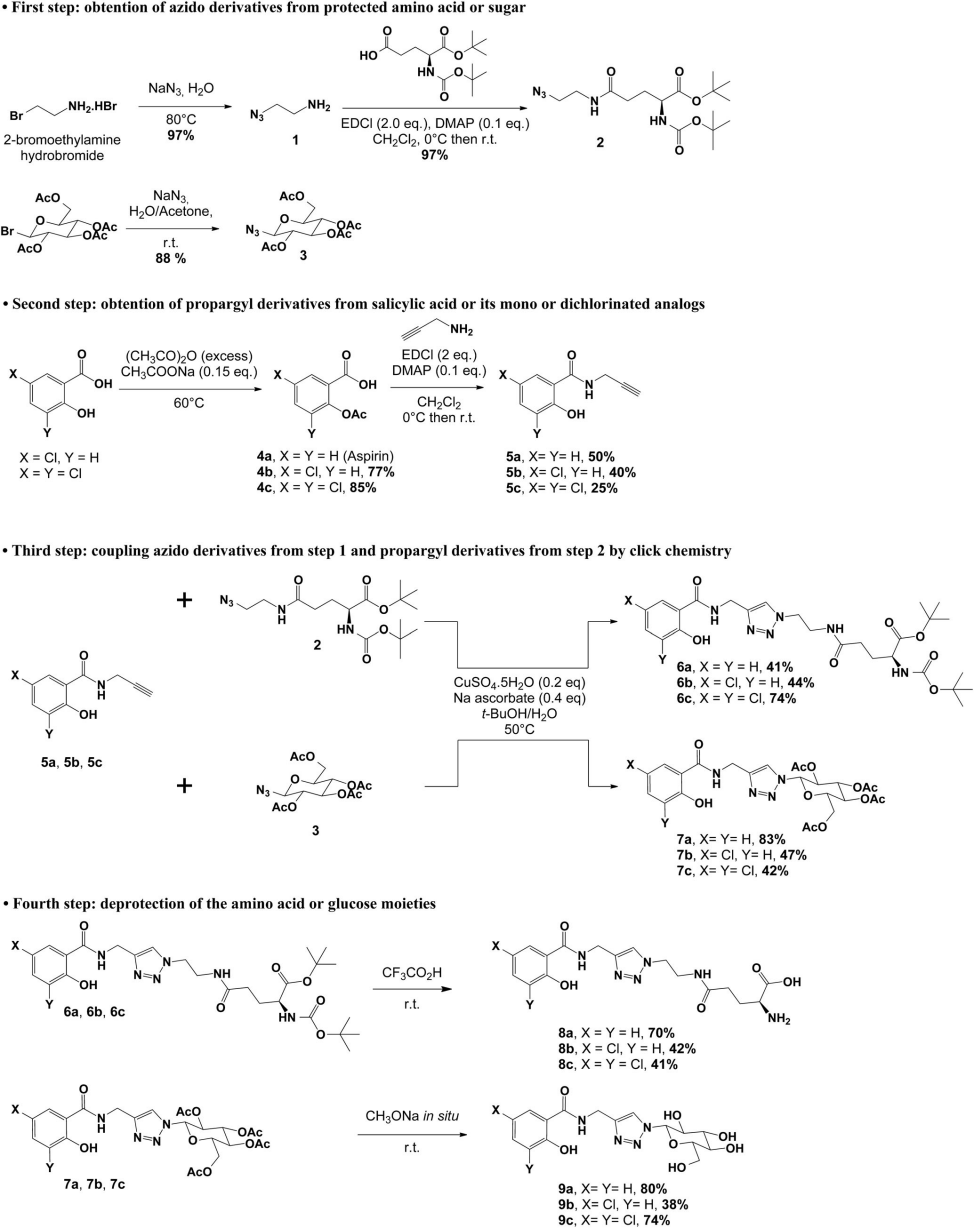
General reaction scheme showing the different steps in the synthesis of amino acid and glucose conjugates of salicylic acid or chlorinated analogs. DMAP: 4‐dimethylaminopyridine; EDCl: 1‐ethyl‐3‐(3‐ dimethylaminopropyl)carbodiimide hydrochloride.

**Figure 2:**
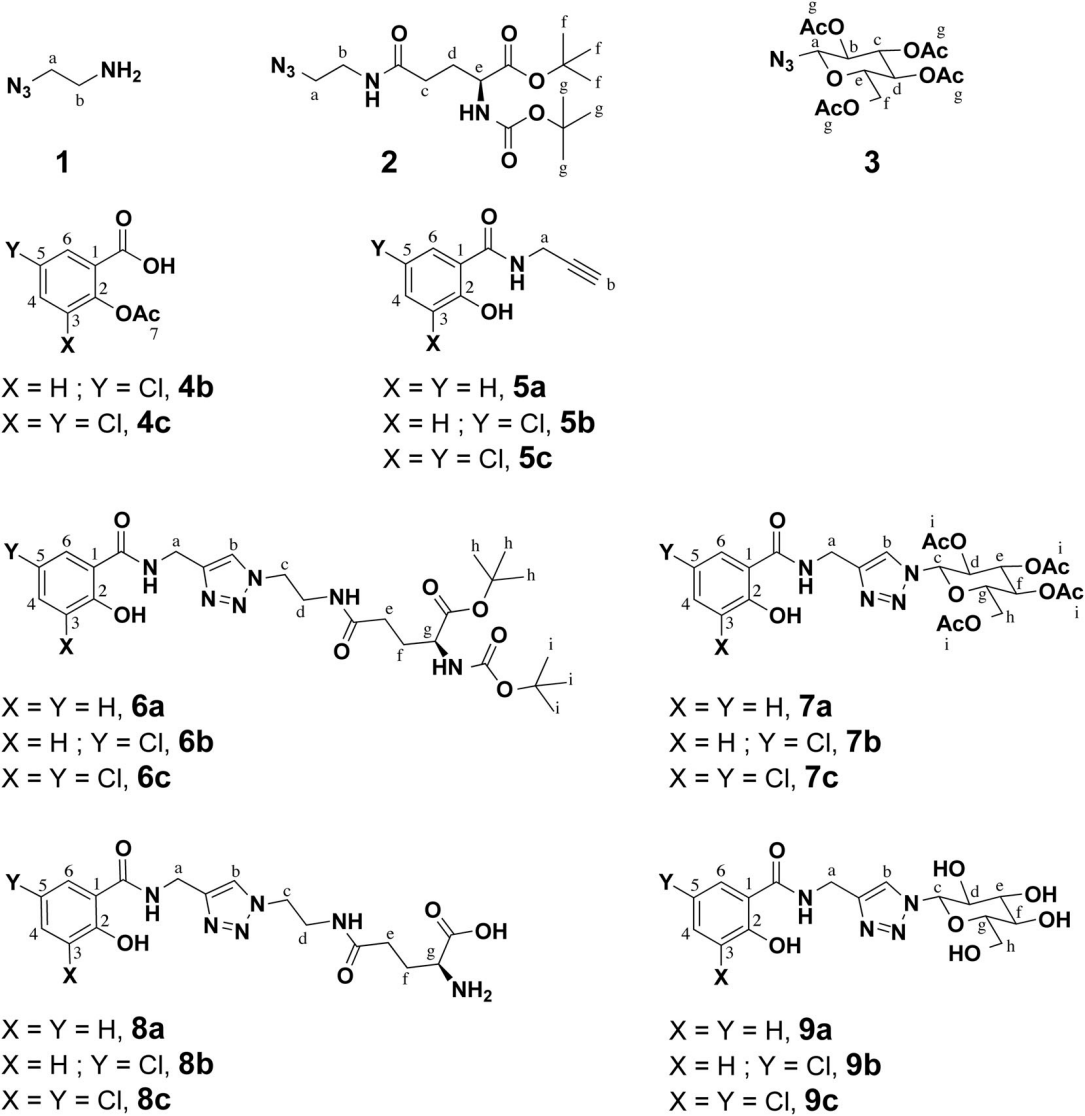
Azido and salicylic acid derivatives numbering for ^1^H and ^13^C assignments.

The azido derivative **2** was prepared from *N*‐Boc‐L‐glutamic acid 1‐*tert*‐butyl ester and 2‐azidoethanamine **1** with a yield of 97% as previously described.^28^

The azido sugar **3** was prepared in 88% yield from 2,3,4,6‐tetra‐O‐acetyl‐α‐*D*‐glucopyranosyl bromide that reacted with sodium azide NaN_3_ *via* a nucleophilic substitution (S_N_2).

##### 3.1.1.1 Experimental procedure for the synthesis of derivative **3**

To a solution of 2,3,4,6‐tetra‐O‐acetyl‐α‐D‐glucopyranosyl bromide (2.02 g, 4.91 mmol, 1 equiv.) in an acetone/water mixture (60:10 mL), sodium azide (1.78 g, 27.4 mmol, 5.6 equiv.) was added. The reaction mixture was stirred at room temperature for 18 h and then acetone was removed under vacuum. The aqueous layer was diluted and extracted three times using ethyl acetate. The combined organic layers were dried over anhydrous MgSO_4_, filtered, and concentrated under vacuum to afford compound **3** as a yellow powder (1.62 g, 4.34 mmol, 88% yield).

##### 3.1.1.2 2,3,4,6‐tetra‐O‐Acetyl‐β‐D‐glucopyranosyl azide (**3**)

Rf = 0.62 (pentane/ethyl acetate 6:4); mp = 128–129 °C (lit. mp = 129 °C). ^1^H NMR (400 MHz, DMSO‐d_6_): δ 5.37 (t, 1H,^3^ *J* =^3^ *J’* = 9.3 Hz, H_c_), 5.18 (d, 1H,^3^ *J* = 9.3 Hz, H_a_), 5.02 (t, 1H,^3^ *J* =^3^ *J’* = 9.3 Hz, H_d_), 4.86 (t, 1H,^3^ *J* =^3^ *J’* = 9.3 Hz, H_b_), 4.2–4.10 (m, 3H, H_e_ and H_f_), 2.08, 2.07, 2.03, 1.99 (4 s, 4 CH_3_, H_g_). ^13^C NMR (101 MHz, DMSO‐d_6_): δ 170.0, 169.5, 169.2, 169.1 (4 CO), 86.2 (CH, C_a_), 72.8 (CH, C_e_), 71.8 (CH, C_c_), 70.2 (CH, C_b_), 67.7 (CH, C_d_), 61.7 (CH_2_, *C*_*f*_), 20.5, 20.3, 20.3, 20.2 (4 CH_3_, C_g_). HRMS (ESI, CH_3_CN): m/z calcd for C_14_H_19_N_3_O_9_ [M + Na]^+^ 396.1019, m/z found 396.1013.

#### 3.1.2 *Synthesis of the propargylic amides of salicylic acid and chlorinated analogues (Figs* 1 *and* 2 *; compounds* ***5a‐c*** *from aspirin and compounds* ***4b‐c*** *)*

The second part of the synthesis was the preparation of salicylic acid alkynes **5a‐c** in a one (**5a**) or two steps (**5b**, **5c**) from acetylated salicylic acid or its halogenated analogues. The first step was the acetylation reaction of 5‐chloro and 3,5‐dichlorosalicylic acids in the presence of a catalytic amount of sodium acetate in acetic anhydride under heating to afford compounds **4b** and **4c** in 77% and 85% yields, respectively. This hydroxyl group protection step was necessary to avoid the formation of the mesomeric form that occurs under basic conditions and significantly reduces the activation ability of (1‐(3‐dimethylaminopropyl)‐3‐ethylcarbodiimide hydrochloride (EDCl) on the carboxylic acid function. Next, aspirin and its chlorinated analogues **4b‐c** were condensed with propargylamine in a basic medium (DMAP) and in presence of the coupling agent EDCl to afford propargylamides **5a‐c** in 50%, 40% and 25% (**c**) yields, respectively. The presence of one or two chlorine atoms was clearly correlated with lower yields, probably due to their electron withdrawing effect, which decreases the reactivity of the carboxylic acid function.

##### 3.1.2.1 Acetylation of 5‐chloro or 3,5‐dichlorosalicylic acids

To a suspension of 5‐chlorosalicylic acid (4.00 g, 23.2 mmol, 1 equiv.) or 3,5‐dichlorosalicylic acid (5.60 g, 27.0 mmol, 1 equiv.) in acetic anhydride (10 mL, 105.8 mmol, 4.6 or 3.9 equiv., respectively), sodium acetate (0.30 g, 3.66 mmol, 0.16 or 0.14 equiv., respectively) was added. The suspension was stirred under heating (60 °C) for 3 h. The mixture was then cooled to room temperature and washed with cold water. The resulting precipitate was isolated on a büchner funnel, washed with cold water and dried in an oven to obtain 2‐(acetyloxy)‐5‐chlorobenzoic acid **4b** (3.85 g, 17.9 mmol, 77% yield) or 2‐(acetyloxy)‐3,5‐dichlorobenzoic acid **4c** (5.75 g, 23.1 mmol, 85% yield).

##### 3.1.2.2 2‐ (Acetyloxy)‐5‐chlorobenzoic acid (**4b**)

White powder; Rf = 0.12 (CH_2_Cl_2_); mp = 142 °C (lit. mp = 141 °C). ^1^H NMR (400 MHz, DMSO‐d_6_): δ 13.44 (br s, 1H, OH), 7.89 (d,^4^ *J* = 2.7 Hz, 1H, H_6_), 7.71 (dd,^3^ *J* = 8.6 Hz,^4^ *J* = 2.7 Hz, 1H, H_4_), 7.26 (d,^3^ *J* = 8.6 Hz, 1H, H_3_), 2.25 (s, 3H, H_7_). ^13^C NMR (101 MHz, DMSO‐d_6_): δ 169.0, 164.5 (2 CO), 148.5 (C, C_2_), 133.5 (CH, C_4_), 130.7 (CH, C_6_), 130.1 (C, C_1_), 125.9 (C, C_5_), 125.9 (CH, C_3_), 20.7 (CH_3_, C_7_).

##### 3.1.2.3 2‐(Acetyloxy)‐3,5‐dichlorobenzoic acid (**4c**)

White powder; Rf = 0.09 (CH_2_Cl_2_); mp = 148 °C. ^1^H NMR (400 MHz, DMSO‐d_6_): δ 13.83 (br s, 1H, OH), 8.06 (d,^4^ *J* = 2.6 Hz, 1H, H_6_), 7.87 (d,^4^ *J* = 2.6 Hz, 1H, H_4_), 2.32 (s, 3H, H_7_). ^13^C NMR (101 MHz, DMSO‐d_6_): δ 168.5, 164.2 (2 CO), 145.7 (C, C_2_), 133.7 (CH, C_4_), 131.2 (C, C_1_), 130.2 (CH, C_6_), 129.6, 127.9 (2 C, C_3_ and C_5_), 20.8 (CH_3_, C_7_).

#### 3.1.3 *Synthesis of alkynes* ***5a‐c*** *from aspirin or its halogenated analogues* ***4b‐c***

##### 3.1.3.1 2‐Hydroxy‐N‐(prop‐2‐yn‐1‐yl)benzamide (**5a**)

To a suspension of acetylsalicylic acid (6.54 g, 36.3 mmol, 1.0 equiv.) in anhydrous methylene chloride (100 mL) at 0 °C and under nitrogen atmosphere, propargylamine (3.99 g, 72.4 mmol, 2.0 equiv.), EDCl (13.88 g, 72.4 mmol, 2.0 equiv.) and DMAP (0.45 g, 3.70 mmol, 0.1 equiv.) were added. The reaction medium was kept under stirring at 0 °C for 1 h, then stirred at room temperature for 23 h. The mixture was washed four times with water and the aqueous layer was extracted once with dichloromethane. The combined organic layers were dried over MgSO_4_, filtered and concentrated under vacuum. The crude product was purified by column chromatography on silica gel using pentane/ethyl acetate (97:3) as eluent to afford **5a** (3.17 g, 18.1 mmol, 50% yield) as a yellowish solid. Rf = 0.55 (CH_2_Cl_2_); mp = 98 °C (lit. mp = 99 °C). ^1^H NMR (500 MHz, DMSO‐d_6_): δ 12.29 (s, 1H, OH), 9.19 (t,^3^ *J* = 5.3 Hz, 1H, NH), 7.84 (dd,^3^ *J* = 8.0 Hz, ^4^ *J* = 1.5 Hz, 1H, H_6_), 7.42 (td,^3^ *J* = 8.5 Hz,^4^ *J* = 1.5 Hz, 1H, H_4_), 6.91 (dd,^3^ *J* = 8.5 Hz,^4^ *J* = 1.5 Hz, 1H, H_3_), 6.89 (td,^3^ *J* = 8.0 Hz,^4^ *J* = 1.5 Hz, 1H, H_5_), 4.10 (dd,^3^ *J* = 5.5 Hz,^4^ *J* = 2.5 Hz, 2H, H_a_), 2.26 (t,^4^ *J* = 2.5 Hz, 1H, H_b_). ^13^C NMR (126 MHz, DMSO‐d_6_): δ 168.5 (CO), 159.7 (C, C_2_), 134.0 (CH, C_4_), 128.1 (CH, C_6_), 118.8 (CH, C_5_), 117.4 (CH, C_3_), 115.1 (C, C_1_), 80.8 (C≡), 73.2 (CH, C_b_), 28.3 (CH_2_, C_a_).

##### 3.1.3.2 5‐Chloro‐2‐hydroxy‐N‐(prop‐2‐yn‐1‐yl)benzamide (**5b**)

Compound **5b** was obtained using the same procedure as described for compound **5a**, except for DMAP which was used at 0.2 equivalent relatively to the product **4b**. The crude product was purified by column chromatography on silica gel gel using pentane/ethyl acetate (19:1) as eluent to afford **5b** (0.79 g, 3.77 mmol, 40% yield) as a white solid. Rf = 0.61 (CH_2_Cl_2_); mp = 167 °C. ^1^H NMR (500 MHz, DMSO‐d_6_): δ 12.27 (s, 1H, OH), 9.22 (t,^3^ *J* = 5.2 Hz, 1H, NH), 7.92 (d,^4^ *J* = 2.7 Hz, 1H, H_6_), 7.46 (dd,^3^ *J* = 8.8 Hz,^4^ *J* = 2.7 Hz, 1H, H_4_), 6.98 (d,^3^ *J* = 8.8 Hz, 1H, H_3_), 4.10 (dd,^3^ *J* = 5.4 Hz,^4^ *J* = 2.5 Hz, 2H, H_a_), 3.20 (t,^4^ *J* = 2.5 Hz, 1H, H_b_). ^13^C NMR (126 MHz, DMSO‐d_6_): δ 166.8 (CO), 158.0 (C, C_2_), 133.4 (CH, C_4_), 127.7 (CH, C_6_), 122.5 (C, C_5_), 119.3 (CH, C_3_), 116.9 (C, C_1_), 80.5 (C≡), 73.4 (CH, C_b_), 28.5 (CH_2_, C_a_).

##### 3.1.3.3 3,5‐Dichloro‐2‐hydroxy‐N‐(prop‐2‐yn‐1‐yl)benzamide (**5c**)

Compound **5c** was obtained using the same procedure as described for compound **5b**, except for reaction conditions where, after 1 h at 0 °C as described above, an additional 5 h reflux heating was required followed by cooling to room temperature overnight. The crude product was purified by column chromatography on silica gel gel using pentane/ethyl acetate (19:1) as eluent to afford **5c** as a white solid in 25% yield. Rf = 0.66 (CH_2_Cl_2_); mp = 182 °C. ^1^H NMR (400 MHz, CDCl_3_): δ 12.35 (s, 1H, OH), 7.49 (d,^4^ *J* = 2.4 Hz, 1H, H_4_), 7.34 (d,^4^ *J* = 2.4 Hz, 1H, H_6_), 6.68 (s, 1H, NH), 4.24 (dd, ^3^ *J* = 5.2 Hz,^4^ *J* = 2.6 Hz, 2H, H_a_), 2.33 (t,^4^ *J* = 2.6 Hz, 1H, H_b_). ^13^C NMR (101 MHz, CDCl_3_): δ 168.1 (CO), 156.1 (C, C_2_), 134.3 (CH, C_4_), 124.4 (C, C_3_ or C_5_), 124.1 (CH, C_6_), 123.5 (C, C_3_ or C_5_), 115.7 (C, C_1_), 78.2 (C≡), 73.0 (CH, C_b_), 29.9 (CH_2_, C_a_).

#### 3.1.4 *Coupling azido derivatives* ***2*** *and* ***3*** *with propargyl derivatives of salicylic acid or its chlorinated analogues* ***5a‐c*** *by click chemistry (Figs* 1 *and* 2 *; compounds* ***6a‐c*** *and* ***7a‐c*** *)*

The third step of the synthesis pathway was to react the azido derivatives **2** and **3** with the propargylic amides **5a‐c** *via* a click chemistry process. This 1,3‐dipolar cycloaddition reaction leads to the introduction of a spacer arm between the parent molecule and the nutrient that includes a 1,2,3‐triazole ring in its structure. The reaction is catalyzed by active Cu(I) generated *in situ* by reducing Cu(II) salts (copper sulfate) with sodium ascorbate in a heated *tert*‐butanol/water medium. These reaction conditions allowed to get specifically the 1,4‐disubstituted regioisomers **6a‐c** and **7a‐c**. The L‐glutamic conjugates **6a‐c** were obtained with 41%, 44% and 74% yields, respectively. The β‐D glucose derivatives **7a‐c** were obtained with 83%, 47% and 42% yields, respectively.

##### 3.1.4.1 *tert‐Butyl 5‐({[4‐[(2‐hydroxybenzamido)methyl]‐1H‐1,2,3‐triazol‐1‐yl]ethyl}amino)‐2‐[(tert‐butoxycarbonyl)amino]‐5‐oxopentanoate* (**6a**)

To a solution of **5a** (3.17 g, 18.1 mmol, 1.0 equiv.) in *tert*‐butanol (100 mL) under nitrogen atmosphere, were added firstly azide **2** (6.72 g, 18.1 mmol, 1.0 equiv.), then copper sulfate pentahydrate (0.90 g, 3.61 mmol, 0.2 equiv.) and sodium ascorbate (1.43 g, 7.22 mmol, 0.4 equiv.) dissolved in water (40 mL). The reaction medium was stirred and heated at 50 °C for 1 h. After cooling to room temperature, the resulting mixture was diluted with water. The aqueous layer was extracted four times with dichloromethane (50 mL) and the combined organic layers were dried over anhydrous MgSO_4_, filtered and concentrated under vacuum. Finally, the crude product was purified by silica gel column chromatography using dichloromethane/ethyl acetate (1:0 then 0:1) to afford **6a** (4.08 g, 7.46 mmol, 41% yield) as a white solid. Rf = 0.48 (EtOAc); mp = 86–87 °C. ^1^H NMR (400 MHz, DMSO‐d_6_): δ 12.46 (s, 1H, OH), 9.32 (t,^3^ *J* = 5.6 Hz, 1H, NH), 8.04 (s, 1H, H_b_), 7.98 (t,^3^ *J* = 5.1 Hz, 1H, NH), 7.87 (dd,^3^ *J* = 7.9 Hz,^4^ *J* = 1.5 Hz, 1H, H_6_), 7.39 (td,^3^ *J* = 7.3 Hz,^4^ *J* = 1.6 Hz, 1H, H_4_), 7.08 (d,^3^ *J* = 7.8 Hz, 1H, NH), 6.91–6.86 (m, 2H, H_3_ and H_5_), 4.53 (d,^3^ *J* = 5.6 Hz, 2H, H_a_), 4.37 (t,^3^ *J* = 6.0 Hz, 2H, H_c_), 3.74 (td,^3^ *J* = 8.0 Hz,^3^ *J* = 5.2 Hz, 1H, H_g_), 3.45 (td,^3^ *J* = 6.0 Hz,^3^ *J* = 5.1 Hz, 2H, H_d_), 2.10 (t,^3^ *J* = 7.7 Hz, 2H, H_e_), 1.85 (td,^2^ *J* = 13.3 Hz, ^3^ *J* = 7.8 Hz,^3^ *J* = 5.2 Hz, 1H, H_f_), 1.67 (td,^2^ *J* = 13.2 Hz,^3^ *J* = 5.2 Hz,^3^ *J* = 7.8 Hz, 1H, H_f_), 1.38, 1.37 (2 s, 18H, H_h_ and H_i_). ^13^C NMR (101 MHz, DMSO‐d_6_): δ 171.7, 171.5, 168.7 (3 CO), 159.9 (C, C_2_), 156.6 (CO), 144.6 (C), 133.8 (CH, C_4_), 127.9 (CH, C_6_), 123.3 (CH, C_b_), 118.6 (CH, C_3_), 117.3 (CH, C_5_), 115.2 (C, C_1_), 80.3, 78.0 (2 C), 59.7 (CH, C_g_), 53.9 (CH_2_, C_c_), 48.6 (CH_2_, C_a_), 34.5 (CH_2_, C_d_), 31.5 (CH_2_, C_e_), 28.6, 27.6 (6 CH_3_, C_h_ and C_i_), 26.4 (CH_2_, *C*_*f*_).

##### 3.1.4.2 *tert‐Butyl 5‐({[4‐[(5‐chloro‐2‐hydroxybenzamido)methyl]‐1H‐1,2,3‐triazol‐1‐yl]ethyl}amino)‐2‐[(tert‐butoxycarbonyl)amino]‐5‐oxopentanoate* (**6b**)

Compound **6b** was synthesized using the same procedure as described for compound **6a**, except for the heating time at 50 °C which has been increased to 3 h. The crude product was purified on silica gel column chromatography using dichloromethane/ethyl acetate (1:0 then 0:1) to afford compound **6b** as a white solid in 44% yield. Rf = 0.52 (EtOAc); mp = 108–109 °C. ^1^H NMR (500 MHz, DMSO‐d_6_): δ 12.45 (s, 1H, OH), 9.37 (t,^3^ *J* = 5.6 Hz, 1H, NH), 8.00 (s, 1H, H_b_), 7.99 (t,^3^ *J* = 5.9 Hz, 1H, NH), 7.96 (d,^4^ *J* = 2.7 Hz, 1H, H_6_), 7.44 (dd,^3^ *J* = 8.8 Hz,^4^ *J* = 2.7 Hz, 1H, H_4_), 7.11 (d,^3^ *J* = 7.8 Hz, 1H, NH), 6.94 (d,^3^ *J* = 8.8 Hz, 1H, H_3_), 4.55 (d,^3^ *J* = 5.6 Hz, 2H, H_a_), 4.39 (t,^3^ *J* = 6.1 Hz, 2H, H_c_), 3.74 (1H, m, H_g_), 3.44 (q,^3^ *J* = 5.9 Hz, 2H, H_d_), 2.10 (t,^3^ *J* = 7.7 Hz, 2H, H_e_), 1.86 (td,^3^ *J* = 7.7 Hz, 1H, H_f_), 1.70 (td,^3^ *J* = 7.7 Hz,^3^ *J* = 4.8 Hz, 1H, H_f_), 1.38 (s, 18H, H_h_ and H_i_). ^13^C NMR (126 MHz, Acetone‐d_6_): δ 173.0, 172.3, 169.7 (3 CO), 161.1 (C, C_2_), 156.4 (CO), 144.9 (C), 134.5 (CH, C_4_), 127.3 (CH, C_6_), 124.1 (CH, C_b_), 123.6 (C, C_5_), 120.4 (CH, C_3_), 116.5 (C, C_1_), 81.4, 79.1 (2 C), 55.0 (CH, C_g_), 50.0 (CH_2_, C_c_), 40.2 (CH_2_, C_a_), 35.8 (CH_2_, C_d_), 32.6 (CH_2_, C_e_), 28.5, 28.1 (6 CH_3_, C_h_ and C_i_), 27.8 (CH_2_, *C*_*f*_).

##### 3.1.4.3 *tert‐Butyl 5‐({[4‐[(3,5‐dichloro‐2‐hydroxybenzamido)methyl]‐1H‐1,2,3‐triazol‐1‐yl]ethyl}amino)‐2‐[(tert‐butoxycarbonyl)amino]‐5‐oxopentanoate* (**6c**)

A synthesis and purification procedure identical to that used for compound **6b** resulted in compound **6c** as a white solid in 74% yield. Rf = 0.58 (EtOAc); mp = 115–116 °C. ^1^H NMR (500 MHz, DMSO‐d_6_): δ 13.63 (s, 1H, OH), 9.71 (t,^3^ *J* = 5.1 Hz, 1H, NH), 8.04 (s, 1H, H_b_), 8.02 (d,^4^ *J* = 2.4 Hz, 1H, H_4_), 7.99 (t,^3^ *J* = 5.5 Hz, 1H, NH), 7.77 (d,^4^ *J* = 2.4 Hz, 1H, H_6_), 7.09 (d, ^3^ *J* = 7.7 Hz, 1H, NH), 4.54 (d,^3^ *J* = 5.5 Hz, 2H, H_a_), 4.36 (t,^3^ *J* = 6.1 Hz, 2H, H_d_), 3.76 (m,^3^ *J* = 5.2 Hz, 1H, H_g_), 3.46 (q,^3^ *J* = 6.0 Hz, 2H, H_d_), 2.10 (t,^3^ *J* = 7.7 Hz, 2H, H_e_), 1.86 (m,^3^ *J* = 7.7 Hz,^3^ *J* = 5.4 Hz, 1H, H_f_), 1.67 (m,^3^ *J* = 7.7 Hz ^*3*^ *J* = 5.3 Hz, 1H, H_f_), 1.39, 1.38 (2 s, 18H, H_h_ and H_i_). ^13^C NMR (126 MHz, DMSO‐d_6_): δ 171.8, 171.5, 168.1 (3 CO), 155.8 (C, C_2_), 155.5 (CO), 143.5 (C), 133.2 (CH, C_b_), 125.8 (CH, C_4_), 123.6 (CH, C_6_), 122.4, 122.0 (2 C, C_3_ and C_5_), 116.3 (C, C_1_), 80.3, 78.0 (2 C), 53.9 (CH, C_g_), 48.7 (CH_2_, C_c_), 38.8 (CH_2_, C_a_), 34.8 (CH_2_, C_d_), 31.5 (CH_2_, C_e_), 28.2, 27.6 (6 CH_3_, C_h_ and C_i_), 26.4 (CH_2_, *C*_*f*_).

##### 3.1.4.4 2‐[4‐[(2‐Hydroxybenzamido)methyl]‐1H‐1,2,3‐triazol‐1‐yl]‐6‐ (ethanoyloxymethyl)tetrahydro‐2H‐pyran‐3,4,5‐triyl triethanoate (**7a**)

To a solution of compound **5a** (1.00 g, 5.71 mmol, 1.0 equiv.) in *tert*‐butanol (25 mL), were added firstly azido β‐D‐glucose **3** (2.15 g, 5.71 mmol, 1.0 equiv.), then copper sulfate pentahydrate (0.28 g, 1.14 mmol, 0.2 equiv.) and sodium ascorbate (0.45 g, 2.27 mmol, 0.4 equiv.) dissolved in water (5 mL). The reaction medium was stirred under heating at 50 °C for 1 h and after cooling to room temperature, diluted with water. The aqueous layer was extracted three times with dichloromethane (50 mL) and the combined organic layers were dried over anhydrous MgSO_4_, filtered and concentrated under vacuum. The crude product was purified by column chromatography on silica gel using a dichloromethane/ethyl acetate mixture (1:1) as eluent to afford compound **7a** (2.60 g, 4.74 mmol, 83% yield) as a yellow solid. Rf = 0.36 (CH_2_Cl_2_/EtOAc 9:1, v/v); mp = 214–216 °C. ^1^H NMR (CDCl_3_, 400 MHz): δ 12.21 (s, 1H, OH), 7.89 (s, 1H, H_b_), 7.45 (d,^3^ *J* = 7.7 Hz, 2H, NH and H_6_), 7.35 (td,^3^ *J* = 8.3 Hz,^4^ *J* = 0.9 Hz, 1H, H_4_), 6.94 (d,^3^ *J* = 8.3 Hz, 1H, H_3_), 6.79 (t,^3^ *J* = 7.4 Hz, 1H, H_5_), 5.85 (d,^3^ *J* = 8.5 Hz, 1H, H_c_), 5.45–5.40 (m, 2H, H_d_ and H_e_), 5.22 (t,^3^ *J* = 9.5 Hz, 1H, H_f_), 4.68 (qd,^2^ *J* = 15.0 Hz,^3^ *J* = 5.0 Hz, 2H, H_a_), 4.28 (dd,^2^ *J* = 12.7 Hz,^3^ *J* = 4.9 Hz, 1H, H_h_), 4.12 (dd,^2^ *J* = 12.6 Hz,^4^ *J* = 2.0 Hz, 1H, H_h_), 3.99 (ddd,^3^ *J* = 10.0 Hz,^3^ *J* = 4.9 Hz,^4^ *J* = 2.0 Hz, 1H, H_g_), 2.06, 2.05, 2.01, 1.83 (4 s, 4 CH_3_, H_i_). ^13^C NMR (CDCl_3_, 101 MHz): δ 170.6, 170.2, 170.1, 169.4, 169.0 (5 CO), 161.6 (C, C_2_), 144.8 (C), 134.5 (CH, C_4_), 126.0 (CH, C_6_), 121.4 (CH, C_b_), 118.8 (CH, C_5_), 118.6 (CH, C_3_), 114.1 (C, C_1_), 85.9 (CH, C_c_), 75.3 (CH, C_g_), 72.7 (CH, C_e_), 70.4 (CH, C_d_), 67.7 (CH, *C*_*f*_), 61.6 (CH_2_, C_h_), 35.0 (CH_2_, C_a_), 20.9, 20.8, 20.7, 20.3 (4 CH_3_, C_i_).

##### 3.1.4.5 2‐[4‐[(5‐Chloro‐2‐hydroxybenzamido)methyl]‐1H‐1,2,3‐triazol‐1‐yl]‐6‐ (ethanoyloxymethyl)tetrahydro‐2H‐pyran‐3,4,5‐triyl triethanoate (**7b**)

Compound **7b** was synthesized using the same procedure as described for compound **7a**, except that azido‐β‐D‐glucose **3** had to be added in slight excess (1.3 equiv.) to alkyne **5b** to complete the reaction. For the same reason, the heating time at 50 °C was extended to 1.5 h. The crude product was purified by column chromatography on silica gel using a dichloromethane/ethyl acetate mixture (0:1 to 4:1) as eluent to afford compound **7b** as a white solid in 47% yield. Rf = 0.39 (CH_2_Cl_2_/EtOAc 9:1, v/v); mp = 245–246 °C. ^1^H NMR (500 MHz, DMSO‐d_6_): δ 12.39 (s, 1H, OH), 9.39 (t,^3^ *J* = 5.7 Hz, 1H, NH), 8.31 (s, 1H, H_b_), 7.90 (d,^4^ *J* = 2.6 Hz, 1H, H_6_), 7.44 (dd,^3^ *J* = 8.8 Hz,^4^ *J* = 2.6 Hz, 1H, H_4_), 6.94 (d,^3^ *J* = 8.8 Hz, 1H, H_3_), 6.30 (d,^3^ *J* = 9.2 Hz, 1H, H_c_), 5.65 (t,^3^ *J* = 9.4 Hz, 1H, H_e_), 5.52 (t,^3^ *J* = 9.5 Hz, 1H, H_d_), 5.15 (t,^3^ *J* = 9.8 Hz, 1H, H_f_), 4.54 (d,^3^ *J* = 5.6 Hz, 2H, H_a_), 4.33 (m, 1H, H_g_), 4.09 (m, 2H, H_h_), 2.00, 1.97, 1.94, 1.77 (4 s, 4 CH_3_, H_i_). ^13^C NMR (126 MHz, DMSO‐d_6_): 170.0, 169.5, 169.3, 168.4, 167.3 (5 CO), 158.4 (C, C_2_), 145.0 (C), 133.4 (CH, C_4_), 127.6 (CH, C_6_), 122.4 (CH, C_b_), 122.1 (C, C_5_), 119.3 (CH, C_3_), 116.8 (C, C_1_), 83.8 (CH, C_c_), 73.2 (CH, C_g_), 72.2 (CH, C_e_), 70.1 (CH, C_d_), 67.5 (CH, *C*_*f*_), 61.8 (CH_2_, C_h_), 34.6 (CH_2_, C_a_), 20.5, 20.4, 20.2, 19.9 (4 CH_3_, C_i_).

##### 3.1.4.6 2‐[4‐[(3,5‐Dichloro‐2‐hydroxybenzamido)methyl]‐1H‐1,2,3‐triazol‐1‐yl]‐6‐ (ethanoyloxymethyl)tetrahydro‐2H‐pyran‐3,4,5‐triyl triethanoate (**7c**)

Compound **7c** was synthesized using the same procedure as described for compound **7b**, but with a heating temperature raised to 60 °C for a six‐hour period in order to have a complete conversion. The purification procedure was also the same as described above for **7b** and this resulted in compound **7c** as a white solid in 42% yield. Rf = 0.43 (CH_2_Cl_2_/EtOAc 9:1); mp = 225–226 °C. ^1^H NMR (DMSO‐d_6_, 500 MHz): δ 13.60 (s, 1H, OH), 9.78 (s, 1H, NH), 8.38 (s, 1H, H_b_), 8.02 (d,^4^ *J* = 1.1 Hz, 1H, H_6_), 7.81(d,^4^ *J* = 1.1 Hz, 1H, H_4_), 6.32 (d,^3^ *J* = 9.3 Hz, 1H, H_c_), 5.67 (t,^3^ *J* = 9.4 Hz, 1H, H_e_), 5.54 (t,^3^ *J* = 9.5 Hz, 1H, H_d_), 5.16 (t,^3^ *J* = 9.8 Hz, 1H, H_f_), 4.56 (d,^3^ *J* = 5.6 Hz, 2H, H_a_), 4.35 (ddd,^3^ *J* = 10.1 Hz,^3^ *J* = 5.4 Hz,^4^ *J* = 2.4 Hz, 1H, H_g_), 4.09 (m, 2H, H_h_), 2.02, 1.99, 1.95, 1.78 (4 s, 4 CH_3_, H_i_). ^13^C NMR (CDCl_3_, 126 MHz): δ 170.7, 170.1, 169.5, 169.2, 168.7 (5 CO), 156.2 (C, C_2_), 144.5 (C), 134.0 (CH, C_4_), 124.5 (CH, C_6_), 124.0, 123.2 (2 C, C_3_ and C_5_), 121.7 (CH, C_b_), 115.8 (C, C_1_), 86.0 (CH, C_c_), 75.3 (CH, C_g_), 72.6 (CH, C_e_), 70.5 (CH, C_d_), 67.7 (CH, *C*_*f*_), 61.5 (CH_2_, C_h_), 35.1 (CH_2_, C_a_), 20.8, 20.7, 20.3 (4 CH_3_, C_i_).

#### 3.1.5 *Deprotection of the amino acid or sugar moieties (Figs* 1 *and* 2 *; compounds* ***8a‐c*** *and* ***9a‐c*** *)*

The last step in the synthesis leading to the desired conjugates is the removal of the protecting groups from the α‐amino acid function on the **6a‐c** products and from the glucose hydroxyl groups on the **7a‐c** products. The *tert*‐butyl protecting groups (ester and carbamate) of the amino acid function of the products **6a‐c** were removed under acidic conditions with trifluoroacetic acid in dichloromethane to afford the final amino acid conjugates **8a‐c** in 70%, 42% and 41% yields, respectively. The acetyl protecting groups of the hydroxyl functions of the β‐D glucose derivatives **7a‐c** were removed with sodium methoxide generated *in situ*. Then, the resulting alkoxides were neutralized using an acidic cation exchange resin (Amberlite IRN 77), giving the final glucose conjugates **9a‐c** in 80%, 38% and 74% yields, respectively.

##### 3.1.5.1 2‐Amino‐5‐[(2‐{4‐[(2‐hydroxybenzamido)methyl]‐1H‐1,2,3‐triazol‐1‐yl}ethyl)amino]‐5‐oxopentanoic acid (**8a**)

To a solution of **6a** (2.04 g, 3.73 mmol) in dry dichloromethane (15 mL), trifluoroacetic acid (7 mL) was added and the mixture was stirred for 3 h at room temperature. After full conversion of **6a**, the reaction mixture was concentrated under vacuum to afford compound **8a** (1.02 g, 2.61 mmol, 70% yield) as a white solid. Rf = 0.11 (EtOAc/CH_3_OH; 19:1); mp = 59–60 °C. ^1^H NMR (500 MHz, DMSO‐d_6_): δ 9.34 (t,^3^ *J* = 5.4 Hz, 1H, NH), 8.15 (t,^3^ *J* = 5.6 Hz, 1H, NH), 7.98 (s, 1H, H_b_), 7.95 (dd,^3^ *J* = 7.8 Hz,^4^ *J* = 1.6 Hz, 1H, H_6_), 7.36 (td,^3^ *J* = 7.5 Hz,^4^ *J* = 1.6 Hz, 1H, H_4_), 6.96 (dd,^3^ *J* = 8.2 Hz,^4^ *J* = 0.8 Hz, 1H, H_3_), 6.85 (td,^3^ *J* = 8.0 Hz, ^4^ *J* = 0.9 Hz, 1H, H_5_), 4.55 (d,^3^ *J* = 5.4 Hz, 2H, H_a_), 4.40 (t,^3^ *J* = 5.6 Hz, 2H, H_c_), 3.39–3.50 (m,^3^ *J* = 5.6 Hz, 2H, H_d_), 3.27 (t,^3^ *J* = 6.0 Hz, 1H, H_g_), 2.10 (m, 2H, H_e_), 1.95 (m, 2H, H_f_). ^13^C NMR (126 MHz, DMSO‐d_6_): δ 172.6, 170.8, 168.1 (3 CO), 159.8 (C, C_2_), 144.8 (C), 133.8 (CH, C_4_), 129.4 (CH, C_6_), 123.9 (CH, C_b_), 118.9 (CH, C_3_), 117.8 (CH, C_4_), 116.8 (C, C_1_), 53.8 (CH, C_g_), 49.0 (CH_2_, C_c_), 39.4 (CH_2_, C_a_), 35.1 (CH_2_, C_d_), 31.8 (CH_2_, C_e_), 27.1 (CH_2_, *C*_*f*_). HRMS (ESI, CH_3_OH): m/z calcd for C_17_H_22_N_6_O_5_ [M + H]^+^ 391.1724, m/z found 391.1739.

##### 3.1.5.2 2‐Amino‐5‐[(2‐{4‐[(3‐chloro‐2‐hydroxybenzamido)methyl]‐1H‐1,2,3‐triazol‐1‐yl}ethyl)amino]‐5‐oxopentanoic acid (**8b**)

Compound **8b** was obtained from its precursor **6b** with the procedure used for product **8a** as a colorless hygroscopic solid in 42% yield. Rf = 0.13 (EtOAc/CH_3_OH; 19:1). ^1^H NMR (500 MHz, DMSO‐d_6_): δ 12.90 (s, 1H, OH), 9.39 (t,^3^ *J* = 5.2 Hz, 1H, NH), 8.14 (t,^3^ *J* = 5.5 Hz, 1H, NH), 8.00 (s, 1H, H_b_), 7.95 (d,^4^ *J* = 2.7 Hz, 1H, H_6_), 7.43 (dd,^3^ *J* = 8.8 Hz,^4^ *J* = 2.6 Hz, 1H, H_4_), 6.99 (d,^3^ *J* = 8.8 Hz, 1H, H_3_), 4.55 (d,^3^ *J* = 5.4 Hz, 2H, H_a_), 4.39 (t,^3^ *J* = 6.0 Hz, 2H, H_c_), 3.71 (t,^3^ *J* = 5.4 Hz, 1H, H_g_), 3.46 (t, ^3^ *J* = 6.0 Hz, 2H, H_d_), 2.18–2.29 (m,^3^ *J* = 6.8 Hz, 2H, H_e_), 1.93 (m,^3^ *J* = 6.8 Hz, 2H, H_f_). ^13^C NMR (126 MHz, DMSO‐d_6_): δ 171.6, 170.9, 166.6 (3 CO), 158.2 (C, C_2_), 144.1 (C), 132.9 (CH, C_4_), 127.7 (CH, C_6_), 123.2 (CH, C_b_), 122.6 (C, C_5_), 119.1 (CH, C_3_), 117.5 (C, C_1_), 51.9 (CH, C_g_), 48.4 (CH_2_, C_c_), 38.7 (CH_2_, C_a_), 34.5 (CH_2_, C_d_), 30.5 (CH_2_, C_e_), 25.8 (CH_2_, *C*_*f*_). HRMS (ESI, CH_3_OH): m/z calcd for C_17_H_21_ClN_6_O_5_ [M + H]^+^ 425.1335, m/z found 425.1336.

##### 3.1.5.3 2‐Amino‐5‐[(2‐{4‐[(3,5‐dichloro‐2‐hydroxybenzamido)methyl]‐1H‐1,2,3‐triazol‐1‐yl}ethyl)amino]‐5‐oxopentanoic acid (**8c**)

Compound **8c** was obtained from the protected parent **6c** with the procedure used for product **8a** as a white hygroscopic solid in 41% yield. Rf = 0.10 (EtOAc/CH_3_OH; 19:1). ^1^H NMR (500 MHz, DMSO‐d_6_): δ 13.63 (s, 1H, OH), 9.73 (t,^3^ *J* = 5.7 Hz, 1H, NH), 8.24 (s, 2H, NH_2_), 8.15 (t,^3^ *J* = 5.6 Hz, 1H, NH), 8.05 (s, 1H, H_b_), 8.02 (d,^4^ *J* = 2.5 Hz, 1H, H_4_), 7.79 (d,^4^ *J* = 2.5 Hz, 1H, H_6_), 4.54 (d,^3^ *J* = 5.6 Hz, 2H, H_a_), 4.38 (t,^3^ *J* = 6.1 Hz, 2H, H_c_), 3.90 (m,^3^ *J* = 5.6 Hz, 1H, H_g_), 3.48 (q,^3^ *J* = 5.9 Hz, 2H, H_d_), 2.37–2.16 (m, 2H, H_e_), 2.02–1.92 (m, 2H, H_f_). ^13^C NMR (126 MHz, DMSO‐d_6_): δ 171.9, 171.3, 168.6 (3 CO), 156.3 (C, C_2_), 144.2 (C), 133.7 (CH, C_6_), 126.2 (CH, C_4_), 124.0 (CH, C_b_), 122.9, 122.6 (2 C, C_3_ and C_5_), 116.8 (C, C_1_), 52.0 (CH, C_g_), 49.1 (CH_2_, C_c_), 40.0 (CH_2_, C_a_), 35.3 (CH_2_, C_d_), 30.9 (CH_2_, C_e_), 26.2 (CH_2_, *C*_*f*_). HRMS (ESI, CH_3_OH): m/z calcd for C_17_H_20_Cl_2_N_6_O_5_ [M + H]^+^ 459.0945, m/z found 459.0940.

##### 3.1.5.4 2‐Hydroxy‐N‐((1‐((2R,3R,4S,5S,6R)‐3,4,5‐trihydroxy‐6‐(hydroxymethyl)tetrahydro‐2H‐pyran‐2‐yl)‐1H‐1,2,3‐triazol‐4‐yl)methyl)benzamide (**9a**)

To a solution of compound **7a** (2.60 g, 4.74 mmol, 1.0 equiv.) in dry methanol (100 mL) at 0 °C under nitrogen, sodium (0.65 g, 28.3 mmol, 6.0 equiv.) was slowly added over 30 min. The reaction mixture was stirred at room temperature for an additional 3 h. The medium was then neutralized by Amberlite 77 resin and filtered through a pad of celite. The celite was washed with methanol and the filtrate was concentrated under vacuum to afford compound **9a** (1.45 g, 3.81 mmol, 80% yield) as a yellow hygroscopic compound. Rf = 0.01 (EtOAc). ^1^H NMR (400 MHz, DMSO‐d_6_): δ 12.49 (s, 1H, OH), 9.47 (t,^3^ *J* = 5.5 Hz, 1H, NH), 8.18 (s, 1H, H_b_), 7.95 (dd,^3^ *J* = 8.0 Hz,^4^ *J* = 1.4 Hz, 1H, H_6_), 7.38 (td,^3^ *J* = 8.5 Hz,^4^ *J* = 1.5 Hz, 1H, H_4_), 6.93 (dd,^3^ *J* = 8.3 Hz,^4^ *J* = 0.7 Hz, 1H, H_3_), 6.86 (td,^3^ *J* = 8.0 Hz,^4^ *J* = 0.7 Hz, 1H, H_5_), 5.50 (d,^3^ *J* = 9.3 Hz, 1H, H_c_), 5.35 (d,^3^ *J =* 5.9 Hz, 1H, OH), 5.26 (d,^3^ *J =* 3.9 Hz, 1H, OH), 5.14 (d,^3^ *J =* 5.9 Hz, 1H, OH), 4.59 (t,^3^ *J =* 4.6 Hz, 1H, OH), 4.55 (d,^3^ *J* = 5.5 Hz, 2H, H_a_), 3.75 (td,^3^ *J* = 9.1 Hz,^3^ *J* = 5.8 Hz, 1H, H_d_), 3.65 (d,^3^ *J* = 10.3 Hz, 1H, H_h_), 3.43 (dd,^2^ *J* = 14.4 Hz,^3^ *J* = 5.7 Hz, 2H, H_f_ and H_h_), 3.37 (m, 1H, H_e_), 3.22 (t,^3^ *J* = 9.1 Hz, 1H, H_g_). ^13^C NMR (100 MHz, DMSO‐d_6_): δ 169.2 (CO), 160.3 (C, C_2_), 144.9 (C), 134.3 (CH, C_4_), 128.5 (CH, C_6_), 122.7 (CH, C_a_), 119.1 (CH, C_5_), 117.8 (CH, C_3_), 115.7 (C, C_1_), 87.9 (CH, C_c_), 80.4 (CH, *C*_*f*_), 77.5 (CH, C_e_), 72.4 (CH, C_d_), 70.0 (CH, C_g_), 61.2 (CH_2_, C_h_), 35.0 (CH_2_, C_a_). HRMS (ESI, CH_3_OH): m/z calcd for C_16_H_20_N_4_O_7_ [M + H]^+^ 381.1405, m/z found 381.1429.

##### 3.1.5.5 5‐Chloro‐2‐hydroxy‐N‐((1‐((2R,3R,4S,5S,6R)‐3,4,5‐trihydroxy‐6‐(hydroxymethyl)tetrahydro‐2H‐pyran‐2‐yl)‐1H‐1,2,3‐triazol‐4‐yl)methyl)benzamide (**9b**)

Compound **9b** was obtained in an identical manner to that used to get **9a**, as a white solid in 38% yield. Rf = 0.01 (EtOAc); mp = 75–76 °C. ^1^H NMR (400 MHz, DMSO‐d_6_): δ 12.46 (s, 1H, OH), 9.42 (t,^3^ *J* = 5,6 Hz, 1H, NH), 8.21 (s, 1H, H_a_), 7.96 (d,^4^ *J* = 2.6 Hz, 1H, H_6_), 7.44 (dd,^3^ *J* = 8.8 Hz,^4^ *J* = 2.6 Hz, 1H, H_4_), 6.95 (d,^3^ *J* = 8.8 Hz, 1H, H_3_), 5.51 (d,^3^ *J* = 9.3 Hz, 1H, H_c_), 5.36 (d,^3^ *J* = 6.0 Hz, 1H, OH), 5.28 (d,^3^ *J* = 4.9 Hz, 1H, OH), 5.16 (d,^3^ *J* = 5.5 Hz, 1H, OH), 4.63 (t,^3^ *J* = 5.6 Hz, 1H, OH), 4.56 (d,^3^ *J* = 5.6 Hz, 2H, H_a_), 3.76 (td,^3^ *J* = 9.1 Hz,^4^ *J* = 6.0 Hz, 1H, H_d_), 3.68 (m, 1H, H_h_), 3.44 (m, 2H, H_f_ and H_h_), 3.35 (m, 1H, H_e_), 3.20 (m, 1H, H_g_).^13^C NMR (101 MHz, DMSO‐d_6_): δ 167.2 (CO), 158.4 (C, C_2_), 144.2 (C), 133.4 (CH, C_4_), 127.7 (CH, C_6_), 122.4 (CH, C_b_), 122.3 (C, C_5_), 119.3 (CH, C_3_), 116.9 (C, C_1_), 87.5 (CH, C_c_), 80.0 (CH, *C*_*f*_), 72.0 (CH, C_d_), 69.6 (CH, C_g_), 65.0 (CH, C_e_), 60.7 (CH_2_, C_h_), 34.7 (CH_2_, C_a_). HRMS (ESI, CH_3_OH): m/z calcd for C_16_H_19_ClN_4_O_7_ [M + H]^+^ 415.1015, m/z found 415.0993.

##### 3.1.5.6 3,5‐Dichloro‐2‐hydroxy‐N‐((1‐((2R,3R,4S,5S,6R)‐3,4,5‐trihydroxy‐6‐(hydroxymethyl)tetrahydro‐2H‐pyran‐2‐yl)‐1H‐1,2,3‐triazol‐4‐yl)methyl)benzamide (**9c**)

Compound **9c** was obtained in an identical manner to that used to get **9a**, as a white solid in 74% yield. Rf = 0.01 (EtOAc); mp = 91–93 °C. ^1^H NMR (400 MHz, DMSO‐d_6_): 13.66 (s, 1H, OH), 9.83 (t,^3^ *J* = 5.3 Hz, 1H, NH), 8.27 (s, 1H, H_b_), 8.08 (d,^4^ *J* = 2.2 Hz, 1H, H_6_), 7.79 (d,^4^ *J* = 2.2 Hz, 1H, H_4_), 5.50 (d,^3^ *J* = 9.3 Hz, 1H, H_c_), 5.35 (d,^3^ *J* = 6.0 Hz, 1H, OH), 5.26 (d,^3^ *J* = 4.8 Hz, 1H, OH), 5.14 (d,^3^ *J* = 5.5 Hz, 1H, OH), 4.57 (m,^3^ *J* = 5.4 Hz, 3H, H_a_ and OH), 3.75 (t,^3^ *J* = 9.1 Hz, 1H, H_d_), 3.68 (dd,^3^ *J* = 10.2 Hz,^3^ *J* = 4.4 Hz, 1H, H_h_), 3.41 (m, 2H, H_f_ and H_h_), 3.37 (t,^3^ *J* = 8.9 Hz, 1H, H_e_), 3.21 (t,^3^ *J* = 9.0 Hz, 1H, H_g_). ^13^C NMR (101 MHz, DMSO‐d_6_): δ 168.7 (CO), 156.2 (C, C_2_), 144.3 (C), 133.7 (CH, C_4_), 126.4 (CH, C_6_), 122.8 (CH, C_a_), 122.8, 122.5 (2 C, C_3_ and C_5_), 116.8 (C, C_1_), 87.9 (CH, C_c_), 80.4 (CH, C_g_), 77.4 (CH, *C*_*f*_), 72.4 (CH, C_d_), 70.0 (CH, C_e_), 61.2 (CH_2_, C_h_), 35.3 (CH_2_, C_a_). HRMS (ESI, CH_3_OH): m/z calcd for C_16_H_18_Cl_2_N_4_O_7_ [M ‐ H]^−^ 447.0474, m/z found 447.0469.

### 3.2 Comparison of the phloem mobility of compounds **8a‐c** and **9a‐c** using the *Ricinus* model

After the cuticle, the plasma membrane is the second physical barrier that must be passed for the uptake and translocation of xenobiotics in plants. Systemic products must cross the cell plasma membrane at least once to enter the conducting cells of phloem.^25^ Therefore, membrane permeation mechanisms are key factors for the long‐distance transport and distribution of xenobiotics in plants. In this context, it became possible to modulate and control the distribution of an active ingredient within the plant by associating it with a vector, which is called the vectorization process. In the case of molecular vectorization used in this work, a vector group (α‐amino acid, sugar) is associated with a biologically active molecule (*i.e*., salicylic acid), allowing the resulting conjugate to be recognized and manipulated by membrane‐based active nutrient transporters.^25^ For example, the accumulation of Lys‐2,4D, a synthesized α‐amino acid conjugate, in roots was 5‐ to 10‐times higher than that of its parent compound 2,4D after foliar application.^24^

**Figure 3:**
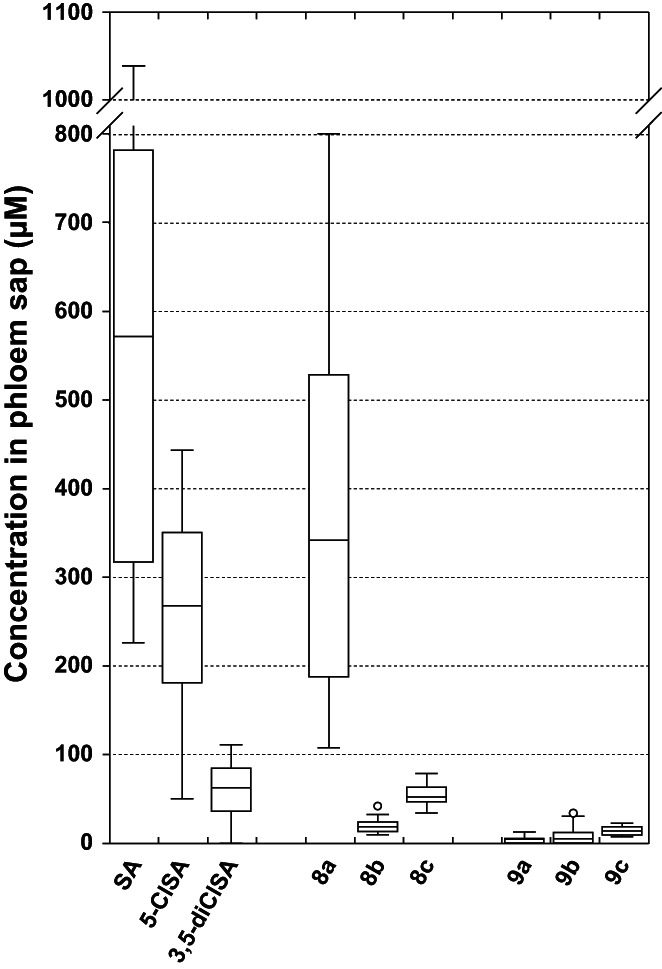
Phloem exudation of salicylic acid, 5‐chlorosalicylic acid (5‐ClSA), 3,5‐dichlorosalicylic acid (3,5‐di‐ClSA) and their amino acid (8a‐c) or glucose conjugates (**9a‐c**) using the *Ricinus* model. After 0.5 h preincubation in the standard medium, the conjugates were added to the incubation medium of cotyledons at 100 μm final concentration, pH 5.0. After 2 h of pre‐treatment, the hypocotyl was severed and the sap was collected from hypocotyl during the third and the fourth hours of cotyledon incubation. For box plots, 8 ≤ n ≤ 16.

Given these considerations, the phloem mobility of conjugates was estimated on the *Ricinus* model, which is a widely used plant model to evaluate phloem systemicity of xenobiotics.^29^ As shown in Fig. 3, SA exhibited very good phloem mobility in *Ricinus* seedling. The concentration factor in phloem sap (the ratio of the concentration in phloem sap/the concentration in the incubation medium) was about 5.7, which was consistent with our previous study (about 6.9).^32^ The phloem mobility of 5‐ClSA and 3,5‐diClSA was much lower than that of SA, showing a concentration factor of 2.7 and 0.6 in phloem sap, respectively. The differences in mobility between the three parent compounds can be attributed to their ability to cross the plasma membrane and to evaluate this, different chemical descriptors or physicochemical properties are commonly used to predict the diffusion of small molecules across human membranes. This approach was later extended to the plant field.^36^ The chemical descriptors that are considered in the Lipinski^37^ (Molecular weight <500 Da; Hydrogen bond donors ≤5; Hydrogen bond acceptors ≤10; Log D ≤ 5.0) or Weber^38^ (Free rotatable bonds ≤10; Polar surface area ≤140 Å^2^) rules differ little between the three compounds, with the exception of the distribution coefficient Log D that increases with the number of chlorine atoms in the molecule (Table 2). For the parent compounds, it is thus clear that their mobility in *Ricinus* is clearly negatively correlated with the number of chlorine atoms in their structure.

Among all of the conjugates, **8a** and **8c** showed almost the same level of phloem mobility with their parent compound, and the concentration factor in phloem sap was 3.4 and 0.52, respectively (Fig. 3). The phloem mobility of glucose conjugates (**9a‐c**) displayed much lower levels (concentration factors 0.04; 0.05; 0.14, respectively) than amino acid conjugates **8a‐c**, suggesting that the amino acid promoiety was more favorable to phloem mobility than that of glucose promoiety. This may be due to the higher substrate specificity of hexose transporters than that of amino acid transporters.^27^, ^28^ Furthermore, it has been shown that SA could be converted in the cytoplasm into several metabolites such as salicylic acid 2‐O‐ß‐D‐glucoside (SAG), which is often the major metabolite. SAG is then compartmentalized in the vacuole by two active transport mechanisms, either through an ATP‐binding cassette transporter mechanism in soybean cells,^39^ either through an H^+^‐antiport mechanism in tobacco cells.^40^ Therefore, it cannot be excluded that the glucose conjugates **9a‐c** were also readily compartmentalized in the vacuole in the same way.

Considering all the conjugates, their chemical descriptors are very similar (Table 2), with the main exception of Log D as before. Contrary to the parent compounds, no correlation between the number of chlorine atoms in the structure and mobility could be observed. Similarly, unlike SA and its chlorinated derivatives, two parameters of Lipinski rule were violated (HBD ≤5; HBA ≤10) as well as one for Veber rule (PSA ≤140 Å^2^). Therefore, the results suggest that these six conjugates are unlikely to diffuse across the plasma membrane and carrier‐mediated processes may contribute to phloem transport, in particular for the conjugate **8a** associating salicylic acid with an amino acid, which shows a remarkable mobility in the *Ricinus* model.

### 3.3 Increased resistance to maize foliar and root pathogen following treatments

**Figure 4:**
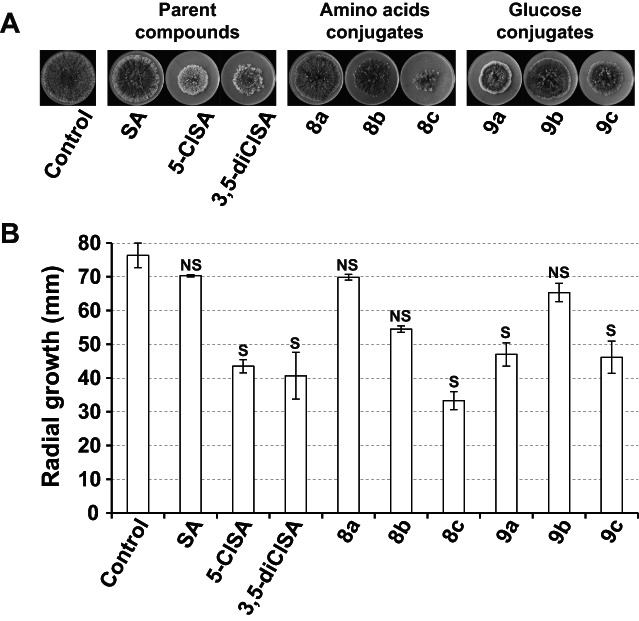
*In vitro* activity of salicylic acid (SA), 5‐chlorosalicylic acid (5‐ClSA), 3,5‐dichlorosalicylic acid (3,5‐diClSA), their amino acid conjugates and their glucose conjugates on the *Bipolaris maydis* mycelial growth. The products were used at 1 mm concentration in the petri dishes and measurements were made 6 days after inoculation. (A), Pictures of a representative experiment. (B), Radial growth measurements of the colonies; n = 3 assays, mean ± CI 95%. The Kruskal‐Wallis test was used to assess statistically significant differences between the control set and the product sets at the 5% probability level. S, significant; NS, non significant.

The *in vitro* and *in vivo* bioassays were conducted to evaluate the fungicidal and defense‐inducing activity of SA conjugates against foliar pathogen *B. maydis*. The mycelial growth of *B. maydis* on the OA plate was not significantly inhibited by SA, amino acid conjugates **8a** and **8b** and glucose conjugate **9b**, indicating that these compounds have no direct fungicidal effect against *B. maydis* at the concentration of 1 mm (Fig. 4). In contrast, the growth of the fungus was inhibited by 43% by 5‐ClSA, 47% by 3,5‐di‐ClSA, 56% by the amino acid conjugate **8c** and 39% by the glucose conjugates **9a** and **9c**. Thus, these products have a direct antifungal activity at 1 mm concentration on the growth of *B. maydis in vitro* (Fig. 4).

**Figure 5:**
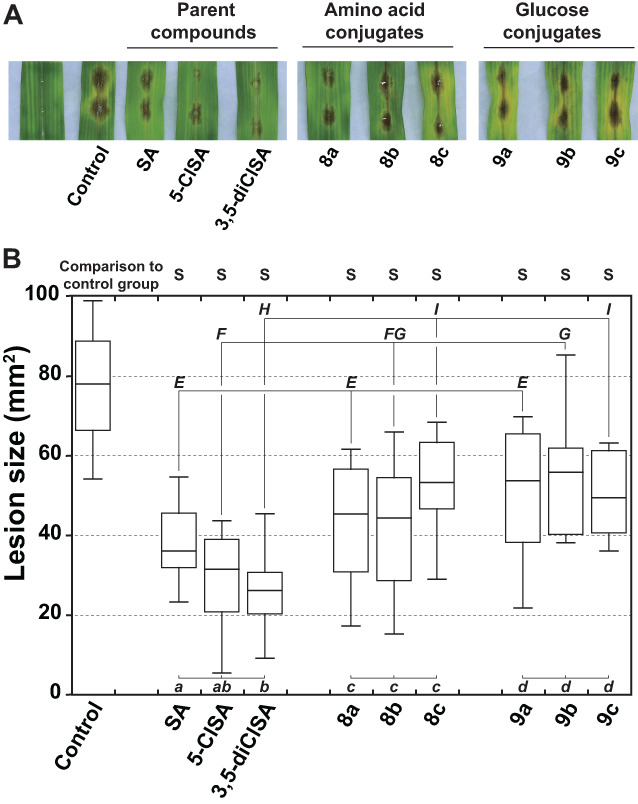
Evaluation of the protective effect of salicylic acid (SA), 5‐chlorosalicylic acid (5‐ClSA), 3,5‐dichlorosalicylic acid (3,5‐diClSA) and their amino acid conjugates (**8a‐c**) and glucose conjugates (**9a‐c**) against *Bipolaris maydis* on maize. Compounds were applied by spraying about 2 mL of a 1 mm concentration solution per plant. After 2 days, maize leaves were harvested and cut into pieces (8 cm). Two droplets of *B. Maydis* spore suspension were applied to the upper surface of each leaf. After 3 days of incubation, all leaves were photographed and lesion size was measured using ImageJ software. (A), Effect of SA conjugates on pathogenicity of *B. maydis* in leaf‐spot inoculation assay. (B), The nine treated groups were compared with the untreated control by performing an ANOVA followed by Tukey's HSD test at the 5% probability level. S: significant. Different lower‐case letters (a, b, c, d) indicated significant differences within each group (parent compounds, amino acid conjugates, glucose conjugates) at the 5% probability level by ANOVA followed by Tukey's HSD test. Different upper‐case letters (E, F, G, H, I) indicated significant differences regarding the chlorinated status of the compounds (0, 1 or 2 chlorine atoms) at the 5% probability level by ANOVA followed by Tukey's HSD test. n = 12 samples.

In *in vivo* experiments, all compounds resulted in significantly increased resistance of maize to prevent *B. maydis* infection when comparing to the ethanol control (Fig. 5). The three parent compounds showed the highest antifungal activity *in vivo*, especially for 3,5‐diClSA exhibiting 66% reduction of the lesion size. The decreasing rates of the lesion size for each conjugate were 42% for **8a**, 43% for **8b**, 32% for **8c**, 31% for **9a**, 28% for **9b** and 37% for **9c**, respectively, which indicated that amino acid or glucose conjugates had almost the same activity level (no significant difference in each separate group; Fig. 5). Conjugates **8a** and **9a** exhibited the same level of antifungal activity with their parent compound SA, demonstrating that the addition of amino acid or glucose promoiety did not affect the defense‐inducing activity of SA against *B. maydis*. However, the lesion size was found to be significantly increased when comparing amino acid conjugate **8c** and glucose conjugates **9b** and **9c** to their corresponding parent compounds 5‐ClSA and 3,5‐diClSA, suggesting that the amino acid or glucose promoiety had negative impact on the activity of chlorinated SA analogues *in vivo*. For example, **8c** showed the highest *in vitro* antifungal activity, but did not exhibit the same trend *in vivo*. It should take into account the possibility that the conjugates cross the plasma membrane into cells easier, then they may not be available in the extracellular spaces where much of the fungal infection would occur. Another possibility would be that the chlorine atoms of the parent compounds reduce the affinity for the active sites of the enzymes that hydrolyze the amido bond of the conjugates, thus affecting the release of the chlorinated analogs of salicylic acid. It has been reported that SA can be conjugated with L‐aspartate (SA‐Asp) as the dominant form in grape (*Vitis vinifera*)^41^ and bean (*Phaseolus vulgaris*).^42^ SA‐Asp is an inactive form of SA,^43^ thus there are certain enzymes existing in plant to release SA from its amino acid conjugates. Future metabolic studies will be conducted to clarify this point.

**Figure 6:**
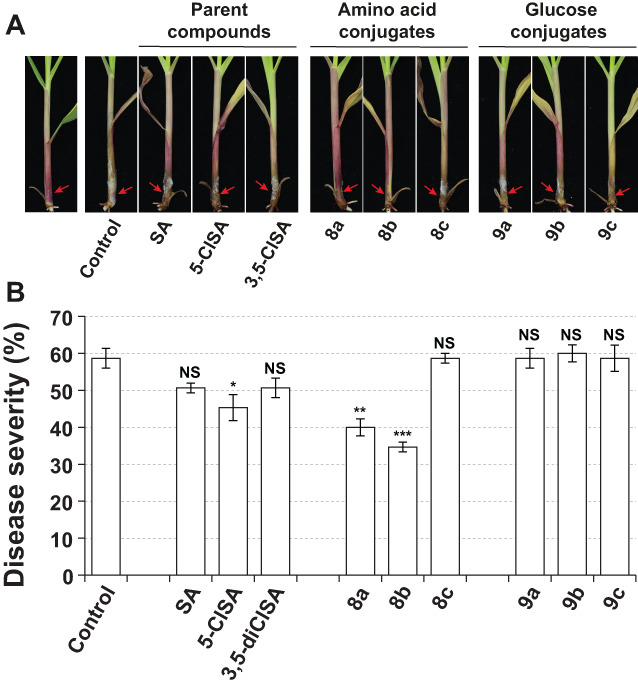
Evaluation of the protective effect of salicylic acid (SA), 5‐chlorosalicylic acid (5‐ClSA), 3,5‐dichlorosalicylic acid (3,5‐diClSA) and their amino acid conjugates (**8a‐c**) and glucose conjugates (9a‐c) against *Fusarium graminearum* on maize. Compounds were applied by spraying about 2 mL of a 1 mm concentration solution per plant. After 2 days, *F. graminearum* conidial suspensions were dropped to the wounded point on the seedling stem (red arrow). (A) Effect of SA conjugates on pathogenicity of *F. graminearum* after artificial inoculation on maize stem. (B) Disease severity in the nine treated groups, which were compared with the untreated control by performing an ANOVA followed by Tukey's HSD Test. Bars represent means ± SE (n = 3 pots of 5 plants). Asterisks indicate significant differences from control (*P < 0.05, **P < 0.01, ***P < 0.001, NS: not significant).

As the same leaves were treated with the tested compound and inoculated with *B. maydis*, it was difficult to discriminate whether the antifungal effect against *B. maydis* was the result of the induced systemic resistance in plants or the direct fungicidal effect of compound. Thus, further bioassays *in vivo* were conducted to investigate the protective effect of SA conjugates on maize stalk rot caused by *F. graminearum* (Fig. 6(A)). The spore suspensions of *F. graminearum* were inoculated on the stem base of maize seedling 2 days after chemical treatment on foliar tissues. As shown in Fig. 6, parent compounds SA and 3,5‐diClSA did not show protect effect against *F. graminearum* under the present experimental conditions, and 5‐ClSA slightly reduced the disease severity of inoculated plants by 13.4%. Conjugates **8a** and **8b** treatment significantly reduced the disease severity by 18.7% and 24.0%, respectively, while there was no beneficial effect for other conjugates (Fig. 6(B)). This is consistent with a previous study in which exogenous application of SA to foliar tissues did not activate defense gene expression in the roots of Arabidopsis^44^ even though SA exhibited very good phloem mobility. This may be due to the fact that SA can be quickly metabolized in plants.^17^ Considering the phloem mobility of the conjugates (Fig. 3), the protective effect of conjugates **8a** and **8b** in the stem could be due to their basipetal transport through phloem. With poor phloem mobility, glucose conjugates **9a‐c** cannot exert their defense‐inducing activity outside the application sites. It is known that biological activity of pesticides can be significantly affected by the uptake and translocation of pesticide within plants, especially for the foliar‐applied pesticides, of which the sites of action may be distant from the point of application.^45^

### 3.4 SA conjugates induced the expression of defense‐related gene *ZmNPR1* and *ZmPR1* upon pathogen challenge

**Figure 7:**
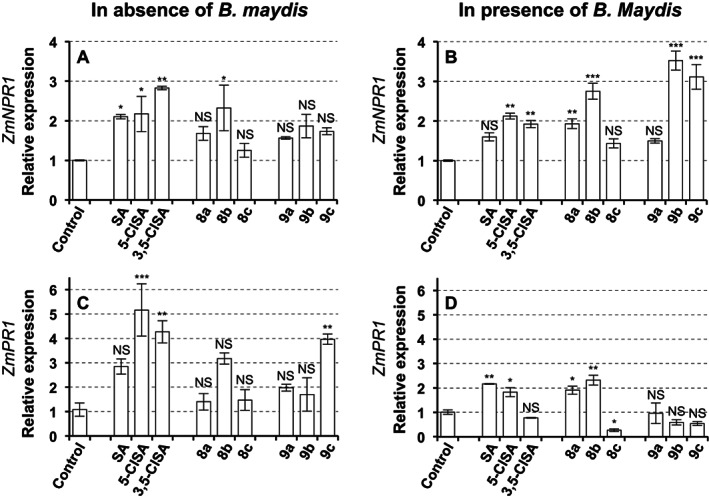
Expression level of defense‐related genes ZmNPR1 (A,B) and ZmPR1 (C, D) in maize leaves treated by SA conjugates and their parent compounds, in the absence (A, C) or in presence (B,D) of *Bipolaris maydis*. The maize seedlings were pretreated with 1 mm SA conjugates or the blank solution (control). After 2 days, spore suspension (1 × 10^5^ conidia/mL) of *B. maydis* was sprayed on the leaves. Samples were harvested at 24 h post‐inoculation. The results were from one representative replicate among three independent experiments showing the same trends. Bars represent means ± SE (n = 3 samples). Asterisks indicate significant differences from control in a Dunnett's multiple comparison test following a one‐way ANOVA (*P < 0.05, **P < 0.01, ***P < 0.001, NS, not significant).

The expression analysis of two defense‐related genes was performed during the plant defense responses following the treatments of SA conjugates. NPR1 and PR1 play important roles in SA signaling pathway in plants. In *Arabidopsis*, NPR1 was found to be the receptor for SA, and the binding of SA to NPR1 was a prerequisite to the transcription of *PR1*.^46^ As shown in Fig. 7, in the absence of pathogen challenge, only parent compounds directly enhanced the expression of *ZmNPR1* and *ZmPR1* 24 h after treatment, while almost no significant change of gene expression was induced by conjugates (Fig. 7(A), (C)). However, following exposure to *B. maydis*, both parent compounds and conjugates increased the expression of *ZmNPR1* and/or *ZmPR1* in maize leaves at 24 h post‐inoculation, demonstrating that SA conjugates were capable of inducing SA‐mediated defense responses upon pathogen attack, except for conjugates **8c** and **9a**. A 3.5‐fold significant increase in *ZmNPR1* expression was observed compared to control after conjugate **9b** treatment (Fig. 7(B)). The plants treated with conjugate **8b** had highest expression (2.3‐fold) of the SA‐responsive marker gene *PR1* (Fig. 7(D)). Amino acid conjugates **8a** and **8b** induced up‐regulation of both genes similar to their parent compounds at 24 h post‐inoculation, while glucose conjugate **9b** and **9c** only increased the expression of *ZmNPR1* showing different patterns with their parent compounds at the same time. These results may be due to the penetration ability of the conjugates across the plasma membrane. The absorption and translocation of glucose conjugates in plant tissues were slower than that of amino acid conjugates according to their physicochemical properties and phloem mobility. Thus, the expression profile induced by glucose conjugates may result from a delayed response.

NPR1 was well known as a key molecule involved in SA signaling, and the *npr1* mutant exhibited enhanced disease susceptibility.^47^ The transcriptional level of *NPR1* increases 2‐ to 3‐ fold following infection with pathogens and SA treatment.^48^ SA and its two halogenated analogues directly triggered stronger defense response than that of their conjugates over short time (24 h) at a concentration of 1 mm. However, conjugates **8a** and **8b** markedly increased the gene expression of two defense‐related genes until the plants were exposed to a challenge infection, suggesting that these conjugates may trigger the plants into a priming phase. Defense priming is a physiological state in which a plant can display longer‐lasting activation or attenuated repression of defense upon pathogen or pest attack than unprimed plants.^49^ SA can directly induce defenses when applied at high doses, but at low doses it can trigger the establishment of defense priming.^50^ Based on prodrug strategy, SA conjugate may act as a novel chemical priming agent, which could be safer to the plant than SA by minimizing fitness costs of resistance.

## 4 CONCLUSION

In conclusion, six conjugates associating salicylic acid, or its analogues monochlorinated in position 5 or dichlorinated in positions 3,5, with glutamic acid or glucose were synthesized, the two moieties being in all cases separated by a spacer arm containing a 1,2,3‐triazole ring. Depending on the conjugates, the syntheses were performed in four to six steps with generally satisfactory yields. Phloem mobility assays using the *Ricinus* model showed that the conjugates with an α‐amino acid promoiety were more favorable to phloem systemicity than those with glucose promoiety. In addition, conjugates **8a** and **8c** retained a concentration factor in the phloem sap of the same order of magnitude as the parent compounds. Conjugate **8a** exhibited the best phloem mobility among all conjugates with a concentration factor of 3.4. Moreover, the amino acid conjugates **8a** and **8b**, as well as the glucose conjugate **9b**, had no direct action on the *in vitro* growth of *B. maydis*, the agent of southern corn leaf blight. In contrast, conjugates **8c**, **9a** and **9c** showed a moderate inhibitory effect on mycelial growth of *B. maydis*. All six conjugates showed a statistically significant protective effect on the size of necroses induced by *B. maydis* on leaves, even the effect appeared to be weaker than the parent compounds especially for the chlorinated molecules. It should be noted that conjugate **8a** reduced necrosis size by 42%, showing similar activity with SA. When tested *in vivo* against *F. graminearum*, conjugates **8a** and **8b** also showed protective effect on stem‐inoculated maize seedlings after foliar application. The expression of defense‐related gene *ZmNPR1* and *ZmPR1* were up‐regulated by SA conjugates upon pathogen challenge. Conjugates **8a** and **8b** increased almost the same level of gene expression as their parent compounds when maize plants were exposed to challenge inoculation with *B. maydis*. Future metabolism studies will be performed to clarify whether SA could be released from the conjugate. Anyway, combining the results of the different bioassays, it seems that the conjugate **8a** is the most promising candidate of this series to stimulate plant defenses, with very good phloem mobility in plant, a preventive effect against pathogens similar to that of salicylic acid and an inducing activity of defense gene at the same level as the parent molecule.

## Data Availability

The data that support the findings of this study are available from the corresponding author upon reasonable request.
